# Automated Detection of External Ventricular and Lumbar Drain-Related Meningitis Using Laboratory and Microbiology Results and Medication Data

**DOI:** 10.1371/journal.pone.0022846

**Published:** 2011-08-02

**Authors:** Maaike S. M. van Mourik, Rolf H. H. Groenwold, Jan Willem Berkelbach van der Sprenkel, Wouter W. van Solinge, Annet Troelstra, Marc J. M. Bonten

**Affiliations:** 1 Department of Medical Microbiology, University Medical Centre Utrecht, Utrecht, The Netherlands; 2 Julius Center for Health Sciences and Primary Care, University Medical Centre Utrecht, Utrecht, The Netherlands; 3 Department of Neurosurgery, University Medical Centre Utrecht, Utrecht, The Netherlands; 4 Department of Clinical Chemistry and Hematology, University Medical Centre Utrecht, Utrecht, The Netherlands; National Institutes of Health, United States of America

## Abstract

**Objective:**

Monitoring of healthcare-associated infection rates is important for infection control and hospital benchmarking. However, manual surveillance is time-consuming and susceptible to error. The aim was, therefore, to develop a prediction model to retrospectively detect drain-related meningitis (DRM), a frequently occurring nosocomial infection, using routinely collected data from a clinical data warehouse.

**Methods:**

As part of the hospital infection control program, all patients receiving an external ventricular (EVD) or lumbar drain (ELD) (2004 to 2009; n = 742) had been evaluated for the development of DRM through chart review and standardized diagnostic criteria by infection control staff; this was the reference standard. Children, patients dying <24 hours after drain insertion or with <1 day follow-up and patients with infection at the time of insertion or multiple simultaneous drains were excluded. Logistic regression was used to develop a model predicting the occurrence of DRM. Missing data were imputed using multiple imputation. Bootstrapping was applied to increase generalizability.

**Results:**

537 patients remained after application of exclusion criteria, of which 82 developed DRM (13.5/1000 days at risk). The automated model to detect DRM included the number of drains placed, drain type, blood leukocyte count, C-reactive protein, cerebrospinal fluid leukocyte count and culture result, number of antibiotics started during admission, and empiric antibiotic therapy. Discriminatory power of this model was excellent (area under the ROC curve 0.97). The model achieved 98.8% sensitivity (95% CI 88.0% to 99.9%) and specificity of 87.9% (84.6% to 90.8%). Positive and negative predictive values were 56.9% (50.8% to 67.9%) and 99.9% (98.6% to 99.9%), respectively. Predicted yearly infection rates concurred with observed infection rates.

**Conclusion:**

A prediction model based on multi-source data stored in a clinical data warehouse could accurately quantify rates of DRM. Automated detection using this statistical approach is feasible and could be applied to other nosocomial infections.

## Introduction

Healthcare-associated infections (HAI) pose a great burden on current medical care and are increasingly viewed as preventable complications. HAI refers to the entire scope of infections associated with medical care and also includes nosocomial infections. The European burden of HAI has been estimated at 4.5 million infections contributing to 148,000 deaths [1]. Hospitals are encouraged to report HAI rates through surveillance organizations such as the National Healthcare Safety Network (NHSN) from the Centers for Disease Control and Prevention (CDC) in the United States [2] and the PREZIES network in the Netherlands [1], [3]. Efficient registration and feedback of infection rates to healthcare workers are considered essential elements to reduce infection rates and surveillance of infection rates is increasingly demanded by policy makers and the public [4]. However, manual registration of HAI rates is time-consuming and susceptible to error due to subjective interpretation of definitions and manual data handling [5], [6].

Therefore, there is an urgent need for more efficient and reliable surveillance methods. Automated classification algorithms using data stored in electronic medical records (i.e. clinical data warehouses [7]) have been developed both at the hospital-wide and procedure-specific level with varying success, in particular for surgical site infections and (catheter-related) bloodstream infections [8]–[18]. Most models use a classification approach based on the presence of (one or more) indicators of infection, such as positive microbiology results, antibiotic use, and discharge coding. Another, less used, method is development of a multivariable model with associated cut-off values to classify patients [11], [13]. Such (automated) models are of relatively low cost, time-saving, and facilitate standardized interpretation of infection criteria [10]. However, when case-finding is based on microbiological cultures, such classification algorithms have low sensitivity for culture-negative infections and specificity of these algorithms decreases when extending case-finding criteria. Furthermore, suboptimal positive predictive values still make manual confirmation of infection necessary. Given these potential drawbacks, most healthcare centers still use manual chart review as their primary method of surveillance.

The aim of this study was to develop a prediction model for HAI using data routinely stored in a clinical data warehouse (i.e. the Utrecht patient-oriented database, UPOD [19]) to retrospectively identify the presence of infection. In order to increase sensitivity for culture-negative infections while maintaining specificity and enable surveillance without the need for manual confirmation, an alternative approach to the classification algorithm was sought and data sources were extended to include not only microbiology results and antibiotic use but also results of clinical chemistry analysis. In clinical practice, such a model could eliminate or significantly reduce the workload of manual chart review and increase resources available for development and implementation of infection control measures. Drain-related meningitis (DRM) was selected as an example to investigate this general approach to automated evaluation of infection rates. This nosocomial infection, related to external cerebrospinal fluid (CSF) drainage through external ventricular (EVD) or lumbar drains (ELD), is sometimes also termed ventriculitis or meningoventriculitis and is one of the procedure-specific infections that has since 2004 been monitored by the department of hospital hygiene and infection control through labor-intensive manual chart review. The developed model achieved good discriminatory power at the level of the individual patient and group-level estimates of infection rates could be generated without any manual confirmation.

## Methods

### Ethics statement

The use of anonymous data through the UPOD has been exempted from review by the Institutional Review Board of the University Medical Center Utrecht as described previously [19].

### Study design and outcome measure

**Figure 1:**
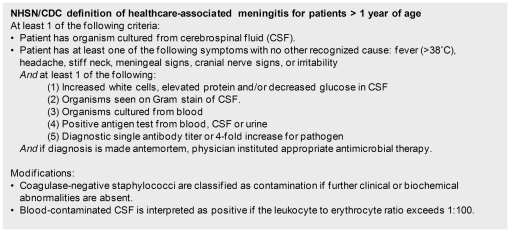
NHSN/CDC definition of healthcare-associated meningitis for patients >1 year of age [**20]**, [21].

Data collected as part of the hospital hygiene surveillance program were used to develop the prediction model for DRM. Results of routinely performed incidence surveys were considered as reference standard. Two infection control professionals assessed each patient for the development of DRM by chart review using modified NHSN/CDC criteria for healthcare-associated meningitis (**figure 1**) [20], [21]; in case of disagreement adjudication was performed through review. A surveillance episode was defined to start the day of drain placement up to seven days after drain removal of the last drain or up to discharge, whichever occurred first.

### Study population

All patients registered by the surveillance program to have received an external cerebrospinal fluid drain at the University Medical Centre Utrecht, a 1042-bed tertiary healthcare centre, were included in this study. Registration comprises all patients who received an EVD between January 1^st^ 2004 and December 31^st^ 2009 (with the exception of May–July 2004), and all patients receiving and ELD in 2004 to 2006. From January 2007 to December 2009 surveillance for ELD was only performed in patients who received the drain in operating theatres. Several infection control measures were implemented during the study period as described previously [20]. All EVDs are placed in operating theatres or, sometimes, under sterile conditions in the intensive care unit (ICU) by a neurosurgeon or trained resident. ELDs are either inserted in the operating theatre or in sterile conditions on the neurology ward. EVDs are tunneled five centimeters under the skin. All patients receive perioperative antibiotic prophylaxis. Drains are not exchanged on a prophylactic basis and CSF samples are collected for culture and biochemical analysis only when infection is clinically suspected; at this time empiric antibiotic therapy is initiated according to local protocol.

Throughout the study period, 742 patients received one or more drains. The following exclusion criteria were applied: death within one day after drain placement (40 patients), age under 18 years (n = 110), pre-existing central nervous system infection (n = 29), more than one simultaneous drain (n = 8), drain placement in a different centre (n = 4), admission duration less than one day (n = 3), second admission more than 30 days after discharge from a first drainage episode within the study (n = 7), and admission to the military hospital (n = 2). After application of exclusion criteria, 537 patients were available for analysis.

### Data collection

The department of hospital hygiene provided outcome data along with the drain characteristics for each patient (drain type, duration, indication for placement). Prediction data was obtained through the Utrecht Patient Oriented Database (UPOD), a clinical data warehouse launched in 2004 for research purposes. The UPOD links results from laboratory analysis (clinical chemistry and hematology), microbiological cultures, and pharmacy dispensing data to information from the hospital admission and discharge system at the patient level [19]. Medication prescription data were obtained both from the UPOD as well as directly from the intensive care unit. Data were adapted to a standardized format and checked for inconsistencies. When necessary, original data sources were cross-referenced to exclude errors.

### Predictor selection

Predictors were selected both on theoretical grounds and to best match the modified NHSN/CDC criteria. Duration of drainage, drain manipulation, subarachnoid hemorrhage, cerebrospinal fluid leakage, and other concomitant infections have been described as possible risk factors for meningitis previously [22]. Besides microbiological analysis of CSF and drains (after removal), biochemical markers of meningitis such as CSF leukocyte count, neutrophil count, glucose level, total protein level, and CSF/blood glucose ratio have been applied to the neurosurgical population with moderate success [23], [24] and were therefore considered as predictors. Unfortunately, Gram-stain results were not yet available and the UPOD does not contain information on drain manipulation and the occurrence of concomitant infections. If patients received both an EVD and an ELD, the EVD took priority in determining drain type. Culture results have been corrected for contamination by categorizing cultures growing coagulase-negative staphylococci as negative if no antibiotic therapy was initiated one day prior through three days after culture. Empiric antibiotic therapy was defined as the simultaneous use of vancomycine and ceftazidime (started four or more days after admission) or ceftriaxone and flucloxacillin (initiated within four days of admission) according to local protocol. The number of systemic antibiotics started throughout the surveillance episode was included as a surrogate marker for the presence of other concomitant infections.

### Statistical analysis

Since the objective was to predict whether a patient had developed DRM during hospital stay, the value that was most indicative of infection measured throughout each patient's surveillance episode was taken for each predictor. Missing data were imputed using multiple imputation (ten imputations). For C-reactive protein (CRP), squared and cubic terms were included in the prediction model along with the linear term. The number of leukocytes in CSF was log-transformed prior to analyses.

Variables were selected for multivariate analysis based on theoretical considerations (as previously described) and results of univariate analysis (p<0.05 in the mean dataset). Using logistic regression analysis, a prediction model was then developed by means of manual backward selection (p<0.05). Although it is recommended to use higher p-values for selection of predictors in prediction research [25], this more stringent criterion was used due to the limited number of events (higher p-values would have resulted in too few events per predictor). Regression coefficients and standard errors were determined on each imputation set and pooled using Rubin's rule [26]. Subsequently, bootstrapping (100 samples per imputation set) was applied to correct for optimism.

Discrimination and calibration were determined for the final model. Discrimination refers to the ability to distinguish between patients with and without DRM; this was assessed by the area under the ROC curve. Calibration refers to the concordance between the predicted and observed probabilities of infection, which was assessed using a calibration plot. For clinical application, cut-off values for a predicted probability associated with high sensitivity and acceptable specificity were determined and associated sensitivity, specificity and predictive values were reported. Confidence intervals were determined using exact binomial methods. Finally, the summed predicted probabilities were used to investigate infection rates at the group level. All analyses were done using SPSS® 17 (SPSS Inc, Chicago IL) and R version 2.11.1 (www.r-project.org).

## Results

A total of 691 drains were placed in 537 patients. DRM occurred in 82 patients (15.3%), or 13.5 infections per 1000 drainage days at risk. The most common causative micro-organisms were coagulase-negative staphylococci (33.8%), followed by *Staphylococcus aureus* (14.6%) and enterobacteriacaea (13.4%). Seventeen infections were culture negative (20.7%). Baseline characteristics are described in **table 1**. Median age of the included patients was 58.5 years, half (n = 263) received a CSF drainage system to treat secondary hydrocephalus following subarachnoid hemorrhage, intraventricular hemorrhage or (cerebellar) infarction and almost 60% (n = 312) of patients were admitted to the ICU during part of their stay. Patients were admitted for a median of 21 days (including readmissions within 30 days).

**Table 1:** Baseline characteristics of the patient population after multiple imputation of missing values and univariate association between variables and the risk of drain-related meningitis.

	Overall	No DRM	DRM	p-value*
Median (IQR) or n (%)	n = 537	n = 455	n = 82	
**Demographics**				
Age (years)	58.5 (47.2–69.6)	59.3 (46.8–69.4)	56.0 (47.5–65.6)	0.49
Sex (% female)	290 (54.0)	247 (54.3)	43 (52.4)	0.78
In-hospital death (%)	90 (16.8)	79 (17.4)	11 (13.4)	0.38
Duration of admission (days)	21.0 (12.0–37.5)	19.0 (11.0–30.0)	40.0 (28.5–59.3)	<0.001
Admission on ICU (%)	312 (58.1)	253 (55.6)	59 (72.0)	0.006
Duration of ICU stay	2 (0.0–7.0)	2 (0.0–5.0)	4.5 (0.0–12.3)	<0.001
Indication for first drain (%)				<0.001
- SAH/IVH	249 (46.4)	205 (42.0)	58 (70.7)	
- Infarction	14 (2.6)	14 (3.1)	0 (0)	
- CSF leakage	85 (15.8)	77 (16.9)	8 (9.8)	
- Perioperative	86 (16.0)	84 (18.5)	2 (2.4)	
- Trauma	14 (2.6)	11 (2.4)	3 (3.7)	
- Tumor	37 (6.9)	30 (6.6)	7 (8.5)	
- Other	52 (9.7)	48 (10.5)	4 (4.9)	
**Drain characteristics**				
Drain type (% EVD)	337 (62.8)	266 (58.5)	71 (86.6)	<0.001
Total drain duration (days)	9.0 (6.0–17.0)	8.0 (5.0–13.0)	20.0 (15.0–29.8)	<0.001
Number of drains placed	1 (1 - 1)	1 (1 - 1)	2 (1–2)	<0.001
**Laboratory measures (blood)**				
CRP (mg/L)	96 (39–173)	85 (32–165)	141 (95–190)	<0.001
Leukocytes (×10^9^/L)	15.7 (11.8–20.1)	14.8 (11.3–19.0)	20.1 (16.3–23.6)	<0.001
Haemoglobin (mmol/L)	6.6 (5.7–7.5)	6.8 (5.8–7.6)	6.0 (5.2–6.8)	<0.001
Thrombocytes (×10^9^/L)	351 (262–495)	329 (252–452)	540 (381–714)	<0.001
**Laboratory measures (CSF)**				
Leukocytes (×100/uL)	1.9 (0.3–5.7)	1.4 (0.2–4.3)	10.4 (2.5–53.1)	<0.001
Erythrocytes (×10000/uL)	1.6 (0.2–7.4)	1.2 (0.2–6.9)	2.4 (0.8–10.6)	0.006
Binary leukocytes (%)	152 (28.3)	91 (20.0)	61 (74.4)	<0.001
Percentage neutrophils	51.7 (33.1–74.0)	47.8 (31.3–66.0)	85.0 (70.0–91.5)	<0.001
Neutrophil count (×100/uL)	0.8 (0.1–4.9)	0.4 (0.0–2.3)	6.3 (0.6–38.0)	<0.001
Glucose (mmol/L)	3.4 (2.7–4.1)	3.5 (2.9–4.2)	2.3 (1.1–3.3)	<0.001
Total protein (g/L)	1.7 (0.8–2.8)	1.7 (0.8–2.7)	1.8 (1.1–3.3)	0.027
**Culture results**				
CSF and/or drain culture (%)	106 (19.7)	45 (9.9)	61 (74.4)	<0.001
**Antibiotic use**				
Any antibiotics started >4 days (%)	271 (50.5)	193 (42.4)	78 (95.1)	<0.001
Any empiric antibiotic therapy (%)	123 (22.9)	61 (13.4)	62 (75.6)	<0.001
Number of antibiotic started	1.0 (0–3)	1 (0–2)	4 (3–6)	<0.001

*: p-value using χ^2^, student's *t* or Mann-Whitney U test where appropriate.

Abbreviations: DRM – drain-related meningitis; IQR –interquartile range; ICU – intensive care unit; CSF - cerebrospinal fluid; EVD – external ventricular drain; HAI – Healthcare-associated infection; SAH – subarachnoid hemorrhage; IVH – intraventricular hemorrhage.

Number of missing values prior to imputation: Other HAI – 37.2%; CRP – 11.2%; Leukocytes (blood) – 8.4%; Hemoglobin – 6.1%; Thrombocytes – 11.2%; CSF leukocytes – 29.2%; CSF erythrocytes 29.1%; CSF glucose 30.7%; CSF protein 29.2%; Culture (CSF and/or drain) – 19.9%. All others: no missing values.

Based on the results of the univariate analysis, the following variables were selected for multivariate analysis: indication for drain placement, duration of admission, number of drains placed, total drainage duration, duration of ICU admission, CRP, blood leukocytes, CSF leukocytes, CSF glucose, CSF protein, culture result (CSF and/or drain), total number of antibiotics started during admission, and whether empiric antibiotic therapy for drain-related meningitis was initiated.

**Figure 2:**
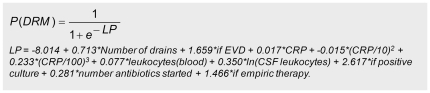
Prediction rule for the development of drain-related meningitis. Abbreviations: P(DRM) – probability of drain-related meningitis; LP – linear predictor; EVD – external ventricular drain; CRP – C-reactive protein; CSF – cerebrospinal fluid.

**Figure 3:**
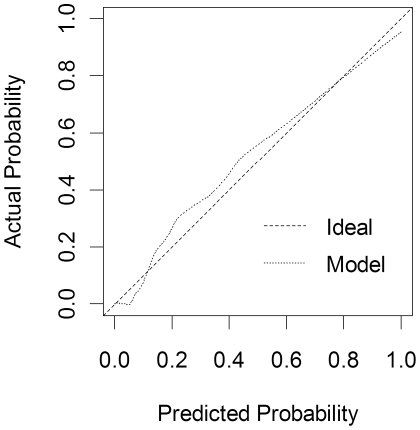
Calibration plot of the model predicting drain-related meningitis. The diagonal dashed line represents ideal prediction by the model, the pointed line predicted probabilities. Calibration, or the concordance between predicted and observed probability of infection, is adequate.

**Table 2** shows the predictors retained in the model and their associated p-values. Despite its high p-value (p = 0.230), the linear CRP term was kept in the model in order to allow the significant high-power terms to be included. The prediction rule can be used to calculate the probability of meningitis for each patient (**figure 2**). Discriminatory power of the model as determined by the area under the ROC curve was 0.970 (95% CI: 0.954–0.986). Calibration of the final model was good (**figure 3**).

**Table 2:** Outcome of backward stepwise logistic regression predicting the risk of drain-related meningitis.

*Predictor*	*OR*	*95% CI*	p-value
Drain type (EVD)	5.26	1.57–17.60	0.003
Number of drains placed	2.04	1.22–3.41	0.005
CRP	1.02	0.99–1.05	0.245
(CRP/10)^2^	0.99	0.97–1.00	0.048
(CRP/100)^3^	1.26	0.99–1.60	0.044
Leukocytes (blood)	1.08	1.01–1.16	0.018
Leukocytes (CSF)	1.42	1.15–1.75	<0.001
Pos culture (drain and/or CSF)	13.70	5.58–33.62	<0.001
Any empiric antibiotics started	1.32	1.04–1.68	0.021
Number of antibiotics started	4.33	1.79–10.5	<0.001

Outcome of backward stepwise logistic regression, cut-off for exclusion p<0.05. Odd's ratio and confidence intervals are after bootstrapping, p-values and predictor selection are prior to bootstrapping and shrinkage. Predictors not retained in model: indication for drain placement, duration of admission, total drainage duration, number of days in intensive care unit, CSF glucose, CSF total protein.

Abbreviations: CI – confidence interval, CRP – C-reactive protein, CSF – cerebrospinal fluid, EVD – external ventricular drain, OR – Odd's ratio.

A cut-off in predicted probability of 0.107 resulted in 98.8% sensitivity, specificity of 87.9% and positive and negative predictive values of respectively 59.6% and 99.8% (**table 3**). The only missed infection was an infection with a coagulase-negative staphylococcus for which no antibiotics were started during admission and of which the patient recovered spontaneously. Selecting a cut-off probability of 0.175 missed three additional infections (sensitivity 95.1%), but only slightly improved specificity (91.0%) and positive predictive value (65.5%).

**Table 3:** Two-by-two contingency table for predicted probability (*P(DRM)*) in relation to drain-related meningitis.

	*DRM*			*Sensitivity*	*Specificity*	*PPV*	*NPV*
Predicted probability	Yes	No	Total	(%)	(%)	(%)	(%)
*P(DRM)*>0.107	81	55	136	98.8	87.9	59.6	99.8
*P(DRM)*≤0.107	1	400	401	(93.4–99.9)	(84.6–90.8)	(50.8–67.9)	(98.6–99.9)
*P(DRM)*>0.175	78	41	119	95.1	91.0	66.5	99.0
*P(DRM)*≤0.175	4	414	418	(88.0–98.7)	(88.0–93.5)	(56.3–74.0)	(97.6–99.7)
Total	82	455	537				

Two-by-two contingency table for predicted probability cut-offs 0.107 and 0.175 in determining the presence of drain-related meningitis with associated sensitivity, specificity, positive and negative predictive values and 95% confidence intervals.

Abbreviations: NPV – negative predictive value, PPV – positive predictive value, P(DRM): predicted probability of drain-related meningitis.

**Figure 4:**
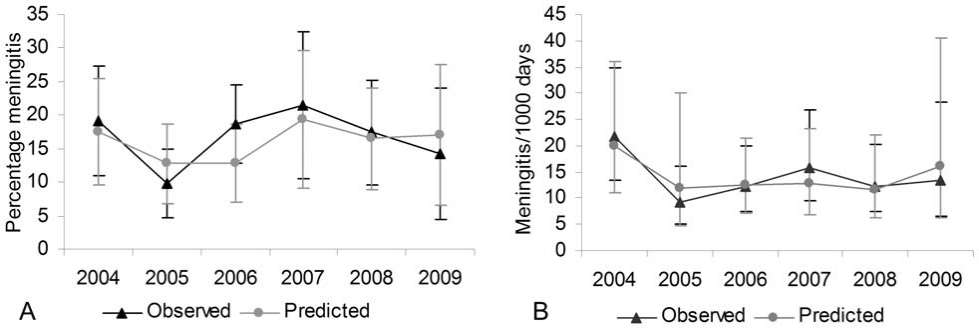
Comparison of observed and predicted overall yearly infection rates. Observed and predicted infection rates at the patient level (panel A) and expressed per 1000 drainage days at risk (panel B), including 95% confidence intervals. Predicted yearly infection rates are determined by the summed predicted probabilities and show good concordance with observed rates.

If a definite diagnosis is necessary at the patient level, application of the model reduced the number of charts to review manually to 25.3% (from 537 to 136 charts) while still identifying 98.8% of infections (81 out of 82). When interested in infection rates at the group level, the summed predicted probabilities reflect total infection percentages with good concordance (**figure 4**) and thus allow for surveillance without the need for manual confirmation.

## Discussion

The results of this study show that information stored in clinical data warehouses can successfully be used to predict rates of DRM in patients receiving an external ventricular or lumbar drain. The combination of drain characteristics, microbiology and clinical chemistry results and antibiotic use achieved 98.8% sensitivity and 87.5% specificity in detecting drain-related meningitis when applying a predicted probability cut-off of 0.107. Negative and positive predictive values were 99.8% and 56.9% respectively. Performing chart review only for those patients identified by the model to have DRM would reduce the number of manual chart reviews by 74.7%. Monitoring of longitudinal infection rates at the group level, however, can be performed without manual confirmation, thereby providing an efficient surveillance tool. This study can be viewed as proof-of-concept for use of regression model-based systems to perform surveillance of nosocomial infections at the group level.

As opposed to most detection models described previously, the model presented here uses data from a multitude of sources in a multivariable model, and case-finding is based on the weighted combination of predictors from each source. As opposed to classification algorithms with case-finding based on broadly selected indicators, this weighted combination of predictors leads to high sensitivity for both culture-positive and culture-negative infections while maintaining acceptable positive predictive value. To the best of our knowledge, this is the first model also incorporating results of clinical chemistry and hematology analysis as predictors of healthcare-associated infections.

In comparison to conventional manual surveillance, this model reduces the time needed to perform surveillance, is less prone to error and less vulnerable to inter-rater variation. Furthermore, calculation of summed predicted probabilities for the at-risk population is an efficient surveillance tool to monitor changes in infection rates and determine when to perform in-depth analysis. Several studies have shown that although automated models using simplified and objective criteria may not always correctly predict absolute infection rates, such models may achieve reliable ranking of hospitals and accentuate differences between hospitals [12], [27], [28].

The large patient population included in this study allowed for the application of statistical methods as opposed to classification algorithms. Although the rule-of-thumb of ten events per predictor was violated, this does not necessarily lead to unreliable results [29]. Furthermore, the selected reference standard, the CDC/NHSN definition of healthcare-associated meningitis, has been measured consistently over time for purposes other than this research. Several other definitions of DRM have been used in literature [22], [30]–[32], however they mostly require positive culture results and therefore have low sensitivity for culture-negative infections which occurred in twenty percent of cases in this population. Even though the CDC/NHSN definition is only partially applicable to neurosurgical patients who are comatose or sedated, the other definitions of DRM will also face this problem as many require the presence of clinical symptoms to confirm the diagnosis. Although it can be argued that healthcare-associated meningitis is a different clinical entity than meningitis secondary to cerebrospinal fluid drainage, the selected reference standard has been measured consistently and reliably over the six-year period and contains many similarities to other definitions proposed for drain-related meningitis. Imputation of missing values was used to prevent the introduction of bias in deriving the model. Since it is not possible to impute missing values for individual patients, a probability of infection can not be computed for future patients with missing data. Out of the patients with an infection, only one had missing data for one predictor (CSF leukocyte count), thereby making underestimation of infection rates unlikely. Furthermore, predictors were only included if commonly determined in clinical practice. For this reason, parameters that have been described previously such as CSF lactate levels [33], [34], CSF cytokine levels [23], [35], and procalcitonin levels [36], [37] were not considered for inclusion. The calculation of the cell-index was considered as a tool to correct for blood-contaminated CSF [38]; however, since this measure could not be calculated in 65.3% of patients due to missing data, it was not included in the analyses. Finally, this model does not investigate infections occurring after discharge unless the patient is readmitted. However, contrary to surgical site infections, post-discharge surveillance is not as relevant since patients often remain in the hospital for a number of days after removal of the drain and it is customary for patients to return to their primary hospital when complications occur. These patients are then re-included in surveillance if readmission occurs within 30 days of discharge.

In summary, the model developed can accurately quantify rates of drain-related meningitis using multi-source data. The proposed model was developed using only retrospective data and although measures have been taken to prevent excessive optimism, prospective validation both within our centre and on a larger scale is necessary to assure applicability to other patient populations. This multivariable model-based approach can be applied to other types of nosocomial infections in the future. Also the development of methods to determine device utilization rates using data available through electronic healthcare records will further improve efficiency and reliability of surveillance.
